# Effects of tDCS on Sound Duration in Patients with Apraxia of Speech in Primary Progressive Aphasia

**DOI:** 10.3390/brainsci11030335

**Published:** 2021-03-06

**Authors:** Charalambos Themistocleous, Kimberly Webster, Kyrana Tsapkini

**Affiliations:** 1 Department of Neurology, Johns Hopkins School of Medicine, Baltimore, MD 21210, USA; cthemis1@jhu.com; 2 Department of Otolaryngology, Johns Hopkins Medicine, Baltimore, MD 21210, USA; kwebste1@jhmi.edu

**Keywords:** apraxia of speech (AOS), transcranial direct current stimulation (tDCS), primary progressive aphasia (PPA), inferior frontal gyrus (IFG), sound duration, brain stimulation

## Abstract

Transcranial direct current stimulation (tDCS) over the left inferior frontal gyrus (IFG) was found to improve oral and written naming in post-stroke and primary progressive aphasia (PPA), speech fluency in stuttering, a developmental speech-motor disorder, and apraxia of speech (AOS) symptoms in post-stroke aphasia. This paper addressed the question of whether tDCS over the left IFG coupled with speech therapy may improve sound duration in patients with apraxia of speech (AOS) symptoms in non-fluent PPA (nfvPPA/AOS) more than sham. Eight patients with non-fluent PPA/AOS received either active or sham tDCS, along with speech therapy for 15 sessions. Speech therapy involved repeating words of increasing syllable-length. Evaluations took place before, immediately after, and two months post-intervention. Words were segmented into vowels and consonants and the duration of each vowel and consonant was measured. Segmental duration was significantly shorter after tDCS compared to sham and tDCS gains generalized to untrained words. The effects of tDCS sustained over two months post-treatment in trained and untrained sounds. Taken together, these results demonstrate that tDCS over the left IFG may facilitate speech production by reducing segmental duration. The results provide preliminary evidence that tDCS may maximize efficacy of speech therapy in patients with nfvPPA/AOS.

## 1. Introduction

Apraxia of speech (AOS) is a condition that affects oral motor speech planning and production. It results in impaired speech fluency due to inhibition of the neural programming of articulation [1]. It can occur in the absence of dysarthria (i.e., a language impairment characterized by paralysis or paresis and muscular control problems) [2] and aphasia (a multimodal language impairment affecting language comprehension and production) [3,4]. Usually, AOS results from stroke, but neurodegeneration, traumatic brain injury, genetic disorders, or syndromes (e.g., childhood apraxia of speech) may also trigger AOS [1,5,6,7,8,9]. In this study, we will refer to AOS in the context of primary progressive aphasia (PPA), a neurodegenerative condition with speech and language deficits as its primary symptoms [10,11,12]. According to the consensus criteria for subtyping of PPA [13], AOS and agrammatism are key symptoms for identifying patients with the non-fluent PPA (nfvPPA) variant from patients with other PPA variants. However, since agrammatism occurs without AOS in some patients [14] and AOS is the only symptom in others [15], a number of studies suggested a clinicopathological presentation of AOS as a distinct PPA variant, primary progressive apraxia of speech (PPAOS) [16,17,18].

The primary characteristics of AOS are articulatory and prosodic deficits with different degrees of severity (mild to severe) [19,20], resulting in effortful, slow speech, manifested by longer consonants and vowels [21,22,23,24]. For example, Duffy and colleagues (2017) [21] argued that slow speech rate and abnormal lexical stress are primary characteristics of progressive AOS that make speech in patients with progressive AOS effortful, slow, labored. Thus, longer segmental duration (vowels and consonants) [25,26] constitute a primary deficit of patients with AOS that distinguishes them from patients with other PPA symptoms [21,22,23,24]. These measures may also serve as an objective and ecologically valid measure of AOS and an excellent outcome measure to estimate the effects of treatment(s) and symptom progression. Patients with AOS exhibit inconsistent and non-systematic speech articulatory errors and irregular insertions, distortions, deletions, substitutions, and transpositions of sounds [27,28,29,30]. They often produce consonants with irregular voicing [31], stop consonants (e.g., /p/ and /t/) with irregular plosive distortions and increased voice onset time (VOT) [24,26,32] or fricative consonants (e.g., /f/ and /θ/) with misplacing and/or misshaping the active articulator (tongue) relative to the passive articulator (a place along the palate) [33]. AOS results in reduced coarticulation of adjacent sounds, a slowing down of syllable transitions, and non-canonical syllable segmentation [26]. Further, irregular prosody and rhythm have been reported as characteristics of speech in patients with AOS [34], affecting lexical (e.g., stress) and post-lexical prominence patterns and tonalities.

AOS symptoms have been associated with the left inferior frontal gyrus (IFG), an area involved in kinematic and sound representations of speech production [6,16,17,35,36]. Patients with AOS show subtle structural and functional irregularities in the IFG, including other areas of the frontal operculum: the posterior frontal gyrus (i.e., pars opercularis [BA44]), which enables the cognitive selection of vocal and orofacial actions [35,37], the pre-supplementary motor area (pre-SMA), which controls vocalization [17], and the insula under the left IFG, which facilitates articulatory planning [20,38]. BA 44 (pars opercularis) is proximal to the premotor cortex, an area involved in articulation and is bi-directionally connected with BA 40 via the ventral component of the superior longitudinal fasciculus (SLF III). This interaction with BA 40 provides BA 44 (which organizes speech production by selecting the phonemes, the words, and their order to form the sentence to be spoken) with critical phonological information. Thus, this cortico-cortical circuit appears to be the phonological loop in the left hemisphere. This parieto-frontal circuit formed by the SLF III (and perhaps the arcuate fasciculus) is involved in phonological processing [39]. The anterior insula is found to be involved in motor speech planning [40]. Other proximal and distal brain regions have also been associated with AOS, such as the parietal lobe [4], the basal ganglia, and the cerebellum [41]. Additionally, there are few behavioral studies with encouraging results targeting AOS symptoms in nfvPPA/AOS [42].

Transcranial direct current stimulation (tDCS) is a non-invasive brain stimulation that modulates neuronal excitability by modifying neural cells’ resting membrane potential either by hyperpolarizing or depolarizing. The placement of the anode (positive electrode) and cathode (negative electrode), intensity, and the duration of stimulation are known to affect the efficacy of tDCS. Recent studies by our group [43] and others [44,45] provided novel insights into the mechanisms of tDCS, showing that changes in functional connectivity (FC) and gamma aminobutyric acid (GABA) concentrations and may be important tDCS mechanisms. We found that tDCS modulated (decreased) functional connectivity (FC) between the stimulated area and the functionally, or structurally, connected temporal areas of the language network, as well as the homologous area in the right hemisphere (but not the default mode network (DMN)); these FC changes were maintained up to 2 months. These results, which are in line with similar decreases in connectivity observed after tDCS over the left IFG in aging [46] and other neurodegenerative conditions, may be interpreted as an indication that fewer resources are needed after tDCS than before for related language tasks. We also tested the hypothesis that tDCS reduces GABA in the stimulated tissue in PPA. We applied GABA-edited magnetic resonance spectroscopy (MRS) to quantify GABA levels before and after a sham-controlled tDCS intervention with language therapy in PPA. Participants receiving tDCS had significantly greater language improvements than those receiving sham immediately after the intervention and at 2 months follow-up. GABA levels in the targeted tissue decreased after the intervention and remained so for 2 months [43].

The association of motor planning and speech articulatory deficits to the left IFG has motivated neuromodulatory studies with tDCS that targeted this area. Specifically, in Marangolo, Marinelli, Bonifazi, Fiori, Ceravolo, Provinciali and Tomaiuolo [19], three subjects with post-stroke aphasia with AOS participated in a randomized double-blinded experiment involving articulatory training in tDCS and sham conditions. Each subject participated in five consecutive daily sessions of anodal tDCS (20 min, 1 mA) and sham stimulation over left IFG. tDCS resulted in more improvement than sham condition. Chesters, et al. [47] tested the effect of tDCS in adults who stuttered and found that anodal tDCS did not improve sentence reading, although, they observed a trend towards a reduction in stuttering when tDCS was coupled with a fluency intervention. In a follow up study, Chesters, et al. [48] tested 30 individuals who stuttered, in which 15 had tDCS and 15 had sham and speech fluency intervention using choral and metronome-timed speech. The authors showed a significant fluency improvement in individuals with tDCS measured one week after the intervention compared to intervention without tDCS. The effects of tDCS were maintained six weeks after therapy during reading but not during conversation. Chester and colleagues concluded that tDCS may be effective for improving speech articulation in other patient populations. Furthermore, the positive effects of tDCS in speech production are supported by studies showing that tDCS improves speech production in typical speakers [49].

Although previous tDCS studies in PPA, including our group’s largest--to our knowledge—double-blind, sham-controlled, cross-over trial of tDCS efficacy in PPA have shown positive effects of tDCS on spoken and written naming and spelling [50,51,52,53,54,55], there is no tDCS study demonstrating the potential of tDCS in reducing AOS symptoms despite the fact that AOS is a prominent feature in patients with nfvPPA or PPAOS (here referred to as nfvPPA/AOS to avoid classification debates). To our knowledge, this is the first study to provide preliminary, proof-of-concept evidence of tDCS efficacy as an adjuvant to speech therapy in PPA patients with AOS symptoms. The present study does not intend to suggest any criterion about AOS diagnosis or test the reliability of perceptual judgments for PPAOS diagnosis (e.g., Dabul et al.’s, questionnaire), or provide evidence for the presence or absence of AOS derived from perceptual judgments. The present study tests a simple hypothesis: if temporal acoustic measures, such as longer sound durations, are a characteristic of AOS [19,20,21,22,23,24,25,26,27], and the left IFG is a critical area for motor planning in speech production [6,16,17,36,37,38,39,40], then tDCS over the left IFG may normalize these sound durations significantly better than speech therapy alone in nfvPPA/AOS.

In the present study, we hypothesized that tDCS over the left IFG coupled with speech production therapy will reduce AOS symptoms in patients with nfvPPA/AOS more than sham, i.e., speech production treatment alone. We used sound duration as a measure of AOS symptoms and reduced sound duration as an improvement of speech production in these patients. As slow speech production is a distinguishing characteristic of speech for patients with nfvPPA/AOS, a decrease in sound duration was considered as a therapeutic improvement corresponding to faster speech articulation. We asked three questions: (1) is tDCS more effective than sham in reducing sound duration in patients with nfvPPA/AOS? (2) Are tDCS effects sustainable over a two-months period? (3) Do tDCS effects generalize to untrained items? To answer these questions, we designed an experimental study where patients with nfvPPA/AOS received anodal tDCS over the left IFG or sham stimulation for the same duration paired with a word repetition task. Patients were evaluated three times: before treatment, immediately after treatment, and two months post-treatment. All words produced were segmented into vowels and consonants and we measured their temporal properties. Changes in syllable length do not affect equally their constituents, namely the vowels and consonants that make up these syllables [26,56,57], as changes in length primarily involve vowels. Therefore, the effects of tDCS on vowel and consonant duration may be different, which motivated us to study the two sound categories separately.

## 2. Materials and Methods

### 2.1. Study Design and Participants

The study had a double-blind, cross-over design with two periods. In the present study, we analyzed only the first period to avoid potential carryover effects. Eight patients with nfvPPA/AOS participated and were recruited from Johns Hopkins clinics or referrals from diagnostic centers. Inclusion criteria were as follows: native English speakers, minimum of high-school education, progressive speech/language disorder diagnosis, and absence of developmental or other neurogenic disorders (e.g., stroke). All participants provided informed consent. We included only those patients with nfvPPA and AOS symptoms (i.e., nfvPPA/AOS). Patients received tDCS or sham for three weeks (15 sessions) and were evaluated three times: before therapy, immediately after therapy, and two months post-therapy. Five participants received anodal tDCS over the left IFG and three participants received sham stimulation, both paired with speech therapy. Patients in the tDCS and sham groups were matched for baseline demographic characteristics and language severity. They were also matched for the segmental duration, which was the dependent variable of the study.

### 2.2. Clinical Assessment

The subtyping of individuals with nfvPPA/AOS followed formal consensus criteria of PPA and was based on cognitive, speech and language testing, neurological examination, and neuroimaging [13]. Table 1 shows the demographic (e.g., age at the beginning of therapy, sex, education) and neuropsychological evaluations for each participant. We report on patients’ performance on the digit span forward and backward, a test measuring short-term and working memory, the Pyramids and Palm Trees [58], a test measuring semantic knowledge, the Boston Naming Test (BNT) [59], a test measuring confrontational naming, and the Subject-relative, Object-relative, Active, and Passive (SOAP), a test for syntactic comprehension [60], and letter and semantic fluency [61]. We also report on disease progression using Fronto-temporal Dementia Clinical Dementia Rating (FTD-CDR) Scale scores for language and total severity (sum of domains) [62]. Severity scores for each domain range from normal (0) to questionable/very mild (0.5), mild (1.0), moderate (2.0), and severe (3.0). Domains included are memory, orientation, judgment and problem-solving, community affairs, home and hobbies, personal care, behavior/comportment, personality, and language [62].

### 2.3. Speech Therapy Methods

Speech therapy was conducted for 45 min total, with tDCS or sham stimulation occurring concurrently for the first 20 min. The therapy task involved oral word repetition of increasingly complex words (e.g., method, methodology, methodological) modeled after Dabul et al.’s standardized assessment [63]. We used ten triplets of increasing morphological complexity for trained words and ten triplets for untrained words matched for frequency, complexity, and length. The trained words were practiced during each therapy session whereas the untrained words were never practiced but were evaluated at all timepoints for both tDCS and sham groups. Patients were initially trained on shorter words and when criterion was met (80% phonetic correctness) they proceeded to the list with increased syllables. The goal was to improve volitional control of participants’ articulators in order to produce co-articulated, intelligible speech, as well as to improve precision of articulation, speech rate, and speech fluency.

### 2.4. tDCS Methods

**Figure 1:**
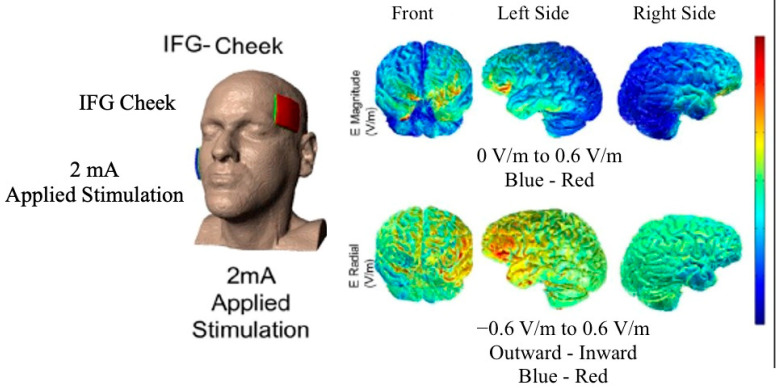
Model of current distribution for used stimulation montage (image courtesy of Dr. Marom Bikson).

To estimate current distribution and guide experimental design, we conducted a current flow analysis for some of our patients of the main trial (see Figure 1), for whom we could obtain those specific scans [64,65]. Stimulation was delivered using the Soterix Transcranial Direct Current Stimulator Clinical Trials Model 1500 at 2 mA intensity for 20 min for a total of 40 mA per session (estimated current density 0.08 mA/cm^2^) [66]. Current was transferred via nonmetallic, conductive rubber electrodes covering 5 × 5 cm (2.54 cm/inch) saline-soaked sponges. The anode was placed over the entire left IFG (see Figure 1) which corresponds to the F7 electrode [53,67,68] based on the electroencephalogram (EEG) 10–20 electrode position system [69]. The left IFG was co-registered to pretreatment magnetic resonance imaging (MRI) scans using a fiducial marker. The cathode was placed on the right cheek. Extracephalic cathodal placement has been shown to better target the area in question (Russell, 2006). Both the participant and the speech-language pathologist were blind to the stimulation condition by means of pre-registered codes on the tDCS device [66]. To mask the condition from participants, sham stimulation involved a short period of electrical current at stimulation onset, ramping up for 30 s and then ramping down, triggering a tingling sensation, which has been shown to blind the participant by creating the same initial sensation as in the tDCS condition [70]. To better simulate the actual tDCS condition during sham condition, we had our device modified to induce a second ramp up and down of the current for 30 s in the middle of the stimulation (about 10 min post-onset) creating an additional short-term tingling sensation to facilitate masking during sham. Patients were debriefed after treatment on whether they received sham or real tDCS and their responses were at chance (53% correct). Participants were asked to report their overall pain level using the Wong–Baker FACES Pain Rating Scale (www.WongBakerFACES.org, accessed on 10 June 2020).

### 2.5. Acoustic Analysis

**Figure 2:**
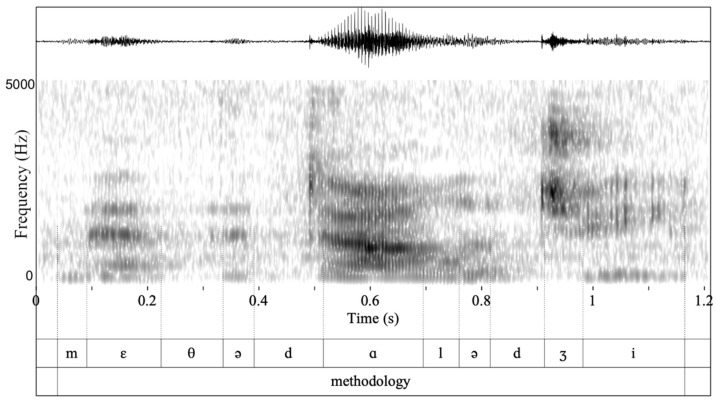
Waveform and spectrogram of the word *methodology*/ˌmɛθəˈdɑləʤi/uttered by a female patient with nfvPPA/AOS. The middle tier shows with thick vertical lines the boundaries of vowels and consonants and the lower shows the target word.

All evaluations (before, immediately after, and 2 months post-therapy) were recorded using an audio recorder that was placed approximately 1 ft in front of the patient. Audio recordings were converted into a 16,000 Hz mono wav file. All word productions were manually split to distinguish the clinician and patient. Figure 2 shows the waveform in the upper tier for the word “methodology”, which served as part of the triplet *method*, *methodology*, *methodological* (see Appendix A for the whole set of words evaluated); the spectrogram is shown under the waveform. The thin vertical lines that extend from the spectrogram to the penultimate tier (measured from top to bottom) indicate the boundaries of vowels and consonants. Each individual sound is denoted in the penultimate tier using the international phonetic alphabet. The whole word is shown in the last tier.

We segmented all individual vowels and consonants uttered by clinicians and patients that made up each keyword as shown in Figure 2 (see also Appendix B, for word characteristics). The segmentation and labeling of vowels and consonants was conducted manually by simultaneous inspection of waveforms and wide-band spectrograms and following standard criteria of segmentation [71]. The onset and offset of the first two vowel formants (F1 and F2) and the fundamental frequency (F0) were employed for the identification of vowels [72,73]. The onset and offset of frication (i.e., the noisy portion) was employed for the identification of fricatives [74,75]. Stop consonants were measured at the onset of the closure phase, including the burst [76]. Segmentation was primarily conducted by the first author and a research assistant. To check for reliability of the segmentation procedures, the first author re-measured 3.5% of the data measured by the research assistant. The duplicate durational measurements of sounds were evaluated using Cohen’s cappa (κ = 0.97, *p* < 0.0001) and show significant agreement. All acoustic analyses were conducted in Praat [71], an acoustic analysis software [77]. From the segmented keywords, we measured the duration of each individual consonant and vowel. To compare consonant and vowel duration between patients and healthy controls, we acoustically analyzed clinicians’ productions, which were provided as prompts in the repetition task during evaluations.

### 2.6. Statistical Analysis

**Figure 3:**
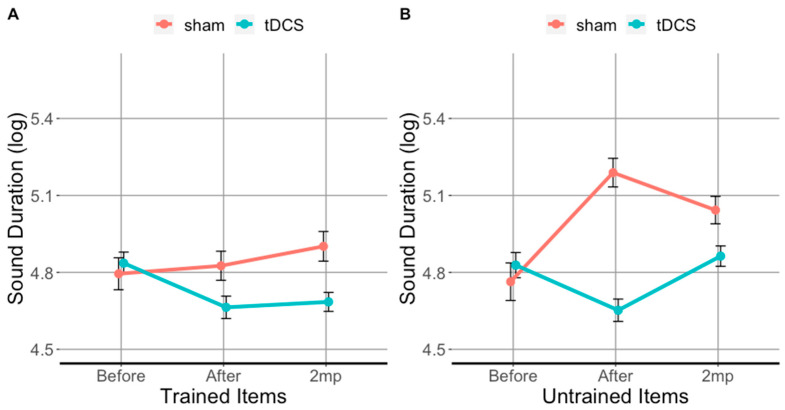
Trained items (**A**) and untrained items (**B**) evaluated before (before), immediately after (after), and 2 months post-treatment (2 mp) for each stimulation condition. The ordinate shows the segmental (vowels and consonants) duration (log transformed), error bars show 95% CI; lower values: shorter segments/faster production. Turquoise lines show tDCS effects; red lines show sham effects.

Patients that received tDCS and sham were matched for segmental duration, the dependent variables of the study, thus they were not different at baseline. To remediate potential confounds due to unequal group sizes, we additionally used a linear mixed effect model that addresses unbalanced designs. We included the participant as a random slope to control for the individual differences between patients, even though the differences in FTD-CDR Total Severity, F.A.S., and SOAP do not reflect on the sound duration at baseline (see Figure 3). Unlike the regression analysis and the analysis of variance (ANOVA), these models incorporate fixed and random effects [78]. The fixed effects are the parameters that we controlled experimentally (stimulation condition and timepoint). The random slope controls for individual differences in the error and increases the robustness of the fixed factors [78,79]. We conducted six linear mixed effects models in R (three for trained and three for untrained items) with the duration of vowels, consonants, and the total sound duration, which pools the duration of vowels and consonants, as dependent variables, and the *condition* (tDCS vs. sham) and *timepoint* (before, after, and two months post-therapy) as predictors. To model individual differences of participants, the *participant* was modelled as a random slope. The linear mixed effects models for trained and untrained items are shown in (1) to (3):

(1) Sound duration ~ condition∗timepoint+(1|participant)

(2) Vowel duration ~ condition∗timepoint+(1|participant)

(3) Consonant duration ~ condition∗timepoint+(1|participant)

Linear mixed effects models were designed in R [80] using the “lme4: Linear Mixed-Effects Models using ‘Eigen’ and S4” package [81], and *p* values were calculated using the LmerTest package [82]. To compute post hoc contrasts, we employed the R package emmeans (EMMs, also known as least-squares means), which provides estimated marginal means [83]. A t test was performed to compare the duration of vowels and consonants produced by patients and clinicians.

## 3. Results

**Figure 4:**
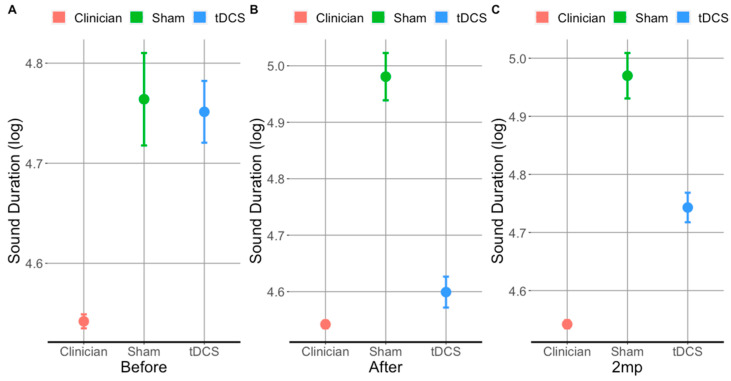
Sound (vowels and consonant) duration (trained and untrained) (log transformed) produced by clinicians and patients that received tDCS and sham evaluated before (**A**), after (**B**), and 2 months post-treatment (**C**). Error bars show 95% CI; lower values: shorter segments/faster production.

At baseline (Figure 4A), the sound duration for trained items did not differ between patients who received tDCS and sham (*t*(3009) = 0.4, *p*= 0.7). Both tDCS and sham patient groups produced significantly longer sounds (trained and untrained) than healthy controls (i.e., the clinicians). However, immediately after treatment (Figure 4B), patients who received tDCS produced significantly shorter sounds than those who received sham (*t*(2508) = 15, *p* < 0.0001), and their sound durations approximated those produced by clinicians (see Figure 4B). Importantly, patients who received tDCS maintained the tDCS-related gains at the 2 months post treatment evaluation for trained items (see Figure 4C). Overall, tDCS resulted in significantly shorter sound durations immediately after and at 2 months post-treatment for both trained and untrained items. We will first present the tDCS vs. sham comparison in trained (i) and untrained items (ii), and then separately for vowels (iii, iv) and consonants (v, vi).

### 3.1. tDCS Effectiveness on Sound Duration in Trained Items

The results for sound duration in the trained items are shown in Figure 3A and Table 2A. Immediately after therapy, sounds in trained items were 26% shorter in the tDCS condition compared to sham. This reduction in sound duration was significant as shown by the post hoc analysis using EMMs (*β =* −0.32, *SE* = 0.04, *df* = 4900.5, *t* = −64.48, *p* = 0.0001). At 2 months post-therapy, sounds in trained words were 29% shorter in tDCS condition compared to sham and the reduction was significant as well (*β* = −0.26, *SE* = 0.05, *df* = 4899.01, *t* = −5.47, *p* = 0.0001). Compared to baseline, sounds in trained words were 19% shorter immediately after therapy (*β* = 0.234, *SE* = 0.035, z(6.800), *p* < 0.0001) and 14% shorter at 2 months post-therapy which is a significant reduction (*β* = 0.234, *SE* = 0.035, *z*(6.800), *p* < 0.0001).

### 3.2. tDCS Effectiveness on Sound Duration in Untrained Items

Figure 3B and Table 2B show the results for sound duration in untrained items. Immediately after therapy, sounds in untrained words were 47% shorter in tDCS condition compared to sham and 22% shorter 2 months post-therapy. Compared to baseline, sounds in untrained words in the tDCS condition were 14% shorter immediately after therapy (*β*= 24, *SE* = 5, *z*(5.100), *p* < 0.0001). However, only a 2% difference in sound duration was observed at 2 months post-therapy for tDCS condition (*β* = 0, *SE* = 5, *z*(0.000), *p =* 1). Compared to baseline, sounds in untrained words in the sham condition were 26% longer immediately after therapy period (*β* = −47, *SE* = 6, *z*(−7.600), *p* < 0.0001) and 19% longer at 2 months post-therapy (*β* = −29, *SE* = 5, *z*(−5.600), *p* < 0.0001).

### 3.3. tDCS Effectiveness on Vowel Duration in Trained Items

**Figure 5:**
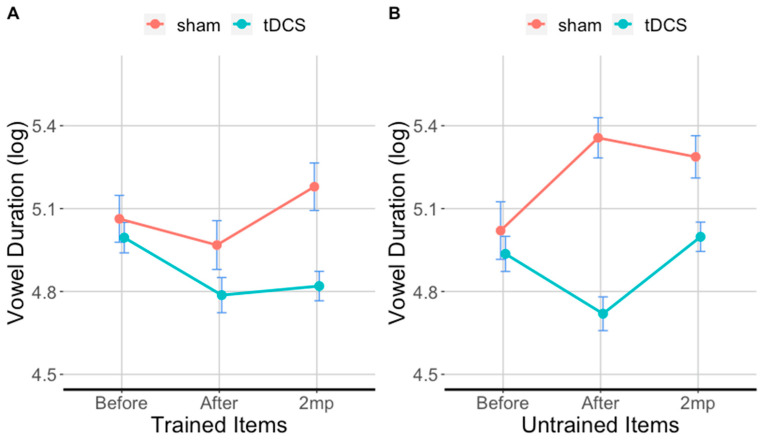
Trained items (**A**) and untrained items (**B**) evaluated before (before), immediately after (after), and 2 months post-treatment (2 mp) for each condition. The ordinate shows vowel duration (log transformed), error bars show 95% CI; lower values: shorter segments/faster production. Turquoise lines show tDCS effects; red lines show sham effects.

Figure 5A and Table 3A show the results for vowel duration in the trained items. Immediately after therapy, vowels in trained words in the tDCS condition were 27% shorter compared to sham (*β* = −0.2434, *SE* = 0.069, *df* = 2043.05, *t* = −3.54, *p* = 0.001). At 2 months post-therapy, vowels in trained words in the tDCS condition were 33% shorter compared to sham (*β* = −0.2820, *SE* = 0.07, *df* = 2041.63, *t* = −4.29, *p* = 0.001). With respect to baseline, vowels in trained words in the tDCS condition were 19% shorter immediately after therapy (*β* = 0.27, *SE*= 0.047, *t =* 5.900, *p* < 0.0001) and 15% shorter 2 months post-therapy (*β* = 0.17, *SE* = 0.041, *t =* 4.200, *p* < 0.0001). No significant change was observed with respect to baseline in sham condition as vowels in trained words were 5.3% longer immediately after therapy (*β* = 0.33, *SE*= 0.219, *t =* 1.500, *p* = 0.68) and 11% longer 2 months post-therapy (*β* = −0.11, *SE* = 0.052, *t =* −2.100, *p* = 0.27).

### 3.4. tDCS Effectiveness on Vowel Duration in Untrained Items

Figure 5B and Table 3B show the results for vowel duration in the untrained items. Immediately after therapy, vowels in untrained words in the tDCS condition were 55% shorter compared sham. They were 30% shorter at 2 months post-therapy compared to sham. With respect to baseline in the tDCS condition, vowels in untrained words were 18% shorter immediately after therapy (*β* = 0.24, *SE* = 0.043, t = 5.500, *p* < 0.0001) and 5% shorter at 2 months post-therapy (*β* = −0.02, *SE*= 0.043, *t* = −0.4, *p* = 1). With respect to baseline in the sham condition, vowels in untrained words were 20% longer immediately after therapy (*β* = −0.37, *SE*= 0.060, *t* = −6.100, *p* < 0.0001) and 17% longer at 2 months post-therapy (*β* = −0.26, *SE* = 0.051, *t* = −5.100, *p* < 0.0001).

### 3.5. tDCS Effectiveness on Consonant Duration in Trained Items

**Figure 6:**
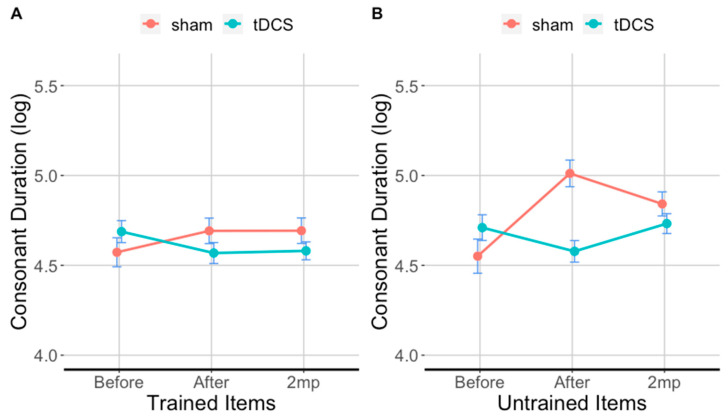
Trained items (**A**) and untrained items (**B**) evaluated before (before), immediately after (after), and 2 months post-treatment (2 mp) for each condition. The ordinate shows consonant duration (log transformed), error bars show 95% CI; lower values: shorter segments/faster production. Turquoise lines show tDCS effects; red lines show sham effects.

Figure 6A and Table 4A show the results for consonant duration in the trained items. Consonants in the tDCS condition were 20% shorter than in the sham condition in the after period, and 17% shorter than sham in the 2 months post speech therapy. With respect to baseline consonants in trained items with tDCS were 15% shorter in the after period (*β* = 0.176, *SE* = 0.046, *t* = 3.8, *p* < 0.0001) 10% shorter in the 2 months post therapy period, an effect that was not significant (*β* = 0.108, *SE* = 0.041, *t* = 2.60 *p* = 0.09). With respect to baseline, consonants in sham condition were only 3.6% longer in the after period (*β* = −0.16, *SE* = 0.045, *t* = −3.400, *p* < 0.01) and 4.3% longer in the 2 months post therapy period (*β* = −0.116, *SE* = 0.045, *t* = −2.6, *p* = 0.1100.

### 3.6. tDCS Effectiveness on Consonant Duration in Untrained Items

Figure 6B and Table 4B show the results for consonant duration in the untrained items. Immediately after therapy, consonants in untrained items in the tDCS condition were 36% shorter compared to sham. At 2 months post-therapy, consonants in untrained items in the tDCS condition were 14% shorter compared to sham. With respect to baseline, consonants in untrained items in the tDCS condition were 10% shorter immediately after therapy, (*β* = 0.13, *SE* = 0.046, *t* = 2.9, *p* < 0.05) and there was a 0% difference at 2 months post-therapy (*β* = −0.03, *SE* = 0.05, *t* = −0.700, *p* = 0.9800). For consonants in untrained items in the sham condition, duration was 30% longer immediately after (*β* = −0.41, *SE* = 0.06, *t* = −7.2, *p* = 0.0001) and 18% longer 2 months post-therapy (*β* = −0.27, *SE* = 0.241, *t* = −1.100, *p* = 0.85) compared to baseline.

## 4. Discussion

In this study, we investigated whether tDCS over the left IFG coupled with speech therapy improves sound duration in patients with nfvPPA/AOS more than sham, i.e., speech therapy alone. First, we evaluated whether tDCS is more effective than sham in improving sound duration in patients with nfvPPA/AOS and whether effects sustained for 2 months post-treatment. Second, we evaluated whether the effects of tDCS generalized to untrained items. Third, we evaluated whether effects differed between vowels and consonants. Our findings show that (1) tDCS in conjunction with speech therapy reduces sound duration significantly more than speech therapy alone (sham). Furthermore, tDCS effects sustained over time, i.e., the tDCS advantage was maintained for up to 2 months post-treatment. (2) The effects of tDCS generalized to untrained items immediately after treatment but this improvement was not maintained at 2 months post-treatment. (3) Patients who received tDCS coupled with speech therapy produced shorter vowels *and* consonants than patients who received speech therapy alone (sham). Below, we discuss the findings in detail, the contribution and limitations of this study, and future directions.

The most important finding of this study is that tDCS reduced sound duration immediately after and up to 2 months post-treatment with respect to baseline for trained and untrained items. Furthermore, in trained items, sound duration approached the sound duration of healthy controls, although sounds produced by patients with nfvPPA/AOS were still significantly longer than those produced by healthy controls. In sham condition, sound duration slightly increased (1.2%) immediately after treatment with respect to baseline and remained the same at 2 months post treatment. This study shows that combining speech training with tDCS induces more sustaining effects. Such sustaining effects of tDCS were observed in other studies related to speech fluency and articulation. For example, Marangolo, Marinelli, Bonifazi, Fiori, Ceravolo, Provinciali and Tomaiuolo [19] also found improvement in response accuracy 2 months post-treatment in three patients with stroke-induced speech apraxia. Chesters, Mottonen and Watkins [48] showed that the tDCS effect on stuttering severity sustained for six weeks post-treatment in reading (but not in conversation). Furthermore, tDCS showed significant generalization of improvement in sound duration relative to sham. Taken together our findings suggest that tDCS has the potential to improve AOS symptoms. This is particularly important for nfvPPA/AOS since some patients may only present with AOS symptomatology at least in initial stages [13,21].

The tDCS montage in the present study targeted the left IFG. As discussed in the Introduction, the left IFG, and in particular the left IFG opercularis, is associated with articulatory motor planning and is adjacent to the primary motor areas of the mouth and tongue [84,85]. Given the size of our electrodes (2 × 2 inches), we cannot claim that we targeted only the left IFG or the IFG opercularis, although this area would be functionally related to AOS symptoms. Recent evidence of the principle of ‘functional targeting’ in the tDCS literature, concurs with the opinion that the current flows only on active cells, those related to the function that is trained [86]. Our previous study has shown that a possible mechanism for tDCS effects is through changes in functional connectivity of the stimulated area, the left IFG, in particular [19]. Although stimulation over the left IFG improved speech production, our findings do not exclude a speech improvement due to stimulation over homologue areas in the right hemisphere or other adjacent areas of the premotor cortex or the insula [87]. A subsequent functional connectivity study would need to provide evidence that this particular stimulation montage caused the present effects of segmental duration of vowels and consonants.

TDCS resulted in shorter vowels and consonants, yet the effects were greater on vowels than consonants. This is not surprising, since vowels and especially stressed vowels, are intrinsically longer than most consonants [22,57], and this is the case even for geminate consonants in languages that have geminates, such as Finnish and Estonian. Therefore, this effect may not reflect a selective effect on vowels but rather opportunities for shortening. There are several underlying causes for these intrinsic differences between vowels and consonants, such as stress, post-lexical prominence (nuclear or pronuclear pitch accents), or phonetic distribution of lengthening over the syllable onset nucleus and coda, which are language specific effects and further discussion would be beyond the scope of this paper. Sound duration is affected by both articulatory and linguistic parameters. Articulatory factors that affect sound duration may be related to articulatory planning, coordination, and timing of neural commands, execution of articulatory movements, control of the airflow from the lungs towards the oral cavity and the vocal fold vibration in the larynx [56,88,89,90,91]. Additionally, phonemic factors that affect sound duration may be related to lexical stress, accentual prominence, lengthening effects demarcating the boundaries of words and phrases, speech fluency, and other communicative effects, such as emphasis [92]. In other words, sound duration is better seen as an integral measure of different processes affecting speech production. The fact that sound duration is improved means that it could be the effect of a multidomain improvement either articulatory or linguistic (lung air pressure, vocal fold vibration, articulatory target approximation, etc.). The additional effects of articulatory deficits in nfvPPA/AOS, may explain why temporal properties of speech have been shown to distinguish patients with AOS from other patients with PPA [8,20,21].

One remaining question is whether tDCS effects transfer to post-lexical coarticulation level phenomena and prosodic phenomena, such as phrasing, intonation, speech fluency, and speech rate that involve post-lexical processes. Word repetition provides very limited information on phrasing, partly because phrasing here would be defined as a measure between clinician-patient-clinician productions (which is partly determined by the clinician). With respect to intonation, it is difficult to study pitch accents (a nuclear pitch accent, a phrase accent, and a boundary tone) at the word level [93]. By studying only F0, it would be very difficult to explain what constitutes an amelioration of the deficit vs. normalization. Furthermore, speech fluency and speech rate require sentence level productions. Nevertheless, segmental duration should be highly correlated with these sentence-level measures, as reduced segmental duration would indicate faster sentence production. Future studies should also incorporate connected speech productions.

The main limitation of this study is the small number of participants, and therefore it can only be considered as a preliminary, proof-of-concept study. A related possible limitation is the matching of participants between the two stimulation groups. We matched the patients with respect to the language component of the FTD-CDR. The participants in the sham group seemed to have a little higher overall severity score, although the difference was not very large (4.67 out of possible 27 in the sham group and 1.5 out of 27 in the tDCS group). The overall severity of the FTD-CDR includes the language component but also provides additional scores for memory, orientation, judgement, community affairs, home and hobbies, personal care, and behavior. Although it is possible to entertain that overall severity differences in other than language sections of the FTD-CDR may impact AOS treatment and tDCS effects, the two stimulation groups were matched at baseline on the AOS outcome measure (sound duration). This, in conjunction with their matched language severity, suggests that the overall severity differences did not affect the outcome measures.

Similarly, patient differences in letter fluency (FAS), and syntactic comprehension (SOAP) were not reflected on sound duration at baseline (the dependent variable of this study) as both groups exhibited approximately the same mean sound duration as shown in Figure 3. If differences in performance on letter fluency or syntactic comprehension tasks influence the neuromodulatory effect of tDCS on sound duration as a primary AOS symptom, it remains an empirical question. Such a finding would rather speak against the consensus classification, i.e., against the fact that nfvPPA is a unitary variant. Rather, it should be split in two as Duffy et al., 2017 have argued: one with AOS symptoms (PPAOS) but without initial fluency or syntactic deficits and another with initial fluency and syntactic deficits and no AOS symptoms. Nevertheless, we acknowledge these differences in the statistics we run, by considering the participant as a random slope.

Another possible limitation is the inherent diffusivity in tDCS methodology, including the lack of specific current flow estimation for each of the present participants. Nevertheless, previous current modeling in Figure 1 showed that the current distribution was centered in the left IFG. The choice for the 5 × 5 cm^2^ electrode patches in our tDCS montage in the present study as well as in most previous clinical studies is driven by the premise and ease of transferring this methodology to clinic, if shown to be efficacious. Although not as precise as other tDCS methodologies, such as high-definition tDCS, the inherent large spread of electrical current in the present and other clinical studies, may actually be the very reason of their efficacy as the current affects larger brain regions. Future studies comparing these methods are needed to determine their clinical efficacy.

## 5. Conclusions

To our knowledge, despite the high prevalence of AOS in PPA, namely nfvPPA, there is no evidence as to whether tDCS may be a useful adjunct to speech therapy in nfvPPA patients with AOS symptomatology. The findings of the present proof-of-concept study, i.e., the remarkable improvement in sound duration immediately after and even up to 2 months post-treatment, shows that tDCS has the potential to enhance speech production in patients with nfvPPA/AOS and warrants a larger study of tDCS over the left IFG as a therapeutic approach to improve AOS symptoms in nfvPPA/AOS. Furthermore, the sustainability of the tDCS’s effects provides the premise that tDCS combined with AOS treatment may inhibit the progression of AOS symptoms in patients with nfvPPA/AOS whose language deteriorates over time due to the nature of neurodegenerative disease. Therefore, a larger behavioral and neuroimaging study is warranted to specifically test the clinical efficacy of tDCS in AOS and the neural structures involved.

## Figures and Tables

**Table 1:** Demographic and neuropsychological data of the participants (numbers out of parenthesis in column mean, indicate the mean and in parenthesis the standard deviation). Total Severity = total severity scale from the Fronto-temporal Dementia Clinical Dementia Rating Scale [62]; Language Severity is the part of the Total Severity FTD-CDR that scores language skills; FAS = The F-A-S Test, a subtest of the Neurosensory Center Comprehensive Examination for Aphasia (NCCEA) [61]; BNT (30) = Boston Naming Test [59]; SOAP Total = Subject-relative, Object-relative, Active, and Passive total score [60]; *p* values are reported from a Kruskal–Wallis rank sum test; * = significant.

	Sham				tDCS						
Participant	ABN	DAN	JJI	Mean	BIN	DRY	GSH	JBN	CDY	Mean	*p*
Education	16	16	16	16 (0)	16	16	20	20	16	18 (2.30)	0.2
Gender	F	F	M	-	M	F	M	M	F	-	
Condition onset (years)	4	2.5	1.5	2.7 (1.3)	3	3.5	6	2	4	3.7 (1.48)	0.2
Age at start of Therapy	54	71	78	67.67 (5.27)	65	53	68	65	74	65 (7.64)	0.5
FTD-CDR Language Severity	2	1	2	1.67(0.58)	1	0.5	2	0.5	1	1(0.6)	0.2
FTD-CDR Total Severity	4	4.5	5.5	4.67 (0.54)	2	0.5	2.5	1	1.5	1.5 (0.79)	0.03 *
F.A.S.	6	11	4	7 (3.51)	21	34	21	31	15	24.4 (7.86)	0.02 *
Fruits, Animals, Vegetables	38	11	10	19.67 (5.32)	33	54	33	42	28	38 (10.27)	0.2
Digit Span Forward	3.5	4	3.5	3.67 (0.25)	4.5	5.5	3.5	6	7	5.3 (1.35)	0.09
Digit Span Backward	2	3.5	2.5	2.67 (0.54)	4.5	5	3.5	3	5.5	4.3 (1.04)	0.07
Pyramids and Palm Trees	15	15	15	15 (0)	15	15	15	15	15	15 (0)	1
BNT (30)	28	28	15	23.67 (6.62)	29	30	24	30	23	27 (3.4)	0.3
SOAP Total (40)	30	33	27	30 (4.24)	35	37	35	33	37	35 (1.7)	0.03 *

**Table 2:** Linear Mixed effects models on the effects of condition (tDCS vs. sham) and period (Before, Immediately After, 2 months post treatment (2 mp)) on trained (top) and untrained sound duration (bottom). The intercept of the model is the value of sham at baseline (Before).

		Estimate	*SE*	*df*	*t*	*p*
**A**. Trained Items	Intercept	4.7955	0.1657	6.1702	28.95	<0.0001
	tDCS vs. sham After	−0.3194	0.0493	4900.5593	−6.48	<0.0001
	tDCS vs. sham at 2 m post	−0.2559	0.0468	4899.0188	−5.47	<0.0001
**B**. Untrained Items	Intercept	4.7427	0.1838	6.12	25.81	0009
	tDCS vs. sham After	−0.59	0.0539	4118.06	−11.02	<0.0001
	tDCS vs. sham at 2 m post	−0.26	0.0495	4113.87	−5.19	<0.0001

**Table 3:** Linear Mixed effects models on the effects of condition (sham vs. tDCS) and period (Before, After, 2 months post therapy (2 mp)) on trained (top) and untrained vowel duration (bottom). The intercept of the model is the value of sham in the before phase.

		Estimate	*SE*	*df*	*t*	*p*
**A**. Trained Items	Intercept	5.0919	0.1728	6.3419	29.47	<0.0001
	tDCS in the After timepoint	−0.2434	0.0687	2043.0476	−3.54	0004
	tDCS in the 2 mp timepoint	−0.2820	0.0657	2041.6251	−4.29	<0.0001
**B**. Untrained Items	Intercept	5.0122	0.1740	6.3172	28.81	<0.0001
	tDCS in the After timepoint	−0.6013	0.0738	1802.2565	−8.15	<0.0001
	tDCS in the 2 mp timepoint	−0.2455	0.0670	1797.7502	−3.66	0002

**Table 4:** Linear mixed effects models on the effects of condition (sham vs. tDCS) and period (BeFigure 2. months post therapy (2 mp)) on trained (top) and untrained consonant duration (bottom). The intercept of the model is the value of sham in the before phase.

		Estimate	*SE*	*df*	*t*	*p*
**A**. Trained Items	Intercept	4.5697	0.1647	6.2897	27.75	<0.0001
	tDCS in the After timepoint	−0.3307	0.0647	2804.4270	−5.11	<0.0001
	tDCS in the 2 mp timepoint	−0.2239	0.0613	2803.4093	−3.65	0.00027
**B**. Untrained Items	Intercept	4.5255	0.1897	6.1737	23.85	<0.0001
	tDCS in the After timepoint	−0.5427	0.0726	2259.5804	−7.48	<0.0001
	tDCS in the 2 mp timepoint	−0.2540	0.0668	2255.8899	−3.80	0.00015

## Data Availability

Data are available after request; sound recordings are not available as they can identify the speaker.

## Appendix A

**Set Lists**	**Word Triplets**		
SET 1	intervene	intervention	interventional
	progress	progression	progressive
	reflect	reflection	reflective
	stimulate	stimulation	stimulating
	stable	stabilize	stabilization
	success	successful	successfully
	excite	excitable	excitability
	improve	improvement	improving
	behave	behavioral	behaviorally
	perform	performance	performing
SET 2	enhance	enhancement	enhancing
	suspend	suspension	suspending
	suppress	suppression	suppressive
	construct	construction	constructive
	accurate	accuracy	inaccurate
	therapy	therapeutic	therapeutically
	provide	provision	provisional
	hypothesis	hypothesize	hypothetical
	define	definition	definitive
	determine	determination	determining
SET 3	inform	information	informative
	suppose	supposition	supposedly
	restrict	restriction	restrictive
	concentrate	concentration	concentrated
	inhibit	inhibition	inhibiting
	investigate	investigation	investigator
	combine	combination	combinatory
	cognition	cognitive	cognitively
	method	methodology	methodological
	courage	courageous	encouraging

## Appendix B

In addition to the quantified measurements, we observed several co-articulatory and phonemic processes that were characteristic of the productions in individuals with nfvPPA/AOS, such as several phenomena that were observed in re-occurring speech of these individuals. Specifically, voiceless consonants were often produced as voiced before voiced nasals (e.g., encouraging /ɪnˈkʰɜrəʤɪn/ > [ɛnˈɡɜːrɪʤɪn], where the vibration of the vocal folds during the production of the nasal/n/does not cease before the production of the adjacent voiceless consonant/k/). Voiced consonants were often produced as devoiced (definitive /dɪˈfɪnɪtɪv/ > [ˌtʰɪvɪnɪˈtʰɪv]; aspiration results from the phonemic rule in English that aspirates onset stop consonants [94]. Overshooting or undershooting of articulatory targets related to the place of articulation (e.g., simulation /ˌsɪmjəˈleɪʃn̩/ > /ˌʃɪmjəˈleɪʃn̩/ an alveolar fricative sound becomes postalveolar fricative when it approaches an alveopalatal consonant). Spirantization phenomena were especially common at word codas (d > ð): method /ˈmɛːθəd/ > [ˈmɛːθəðə]. Affrication of a stop that is produced as fricative or affricate (interventional /ˌɪntərˈvɛnʧənəl/ > [iʰntʃʰ.ɪntʃʰɝ.ˈ væ.stɔ.nəl]). Lastly, other coarticulatory phenomena were also observed, such as cluster simplification: (the/st/): stable/ˈsteɪbl̩/ > /ˈseɪbə/; omission: (the aspiration/h/): enhancement /ɛnˈhænsmənt/ > /ɛnˈæsməns/ here the articulatory command for its production fails to activate). Compensatory measures to produce lexical stress: i. longer syllable duration; ii. splitting the stressed syllable from the preceding part: excitability /ɪkˌsaɪtəˈbɪlɪti/ > [ɪkˌsaɪtə.ˈbɪlɪti]. Slow speech production and effortful speech. To explain the complex interactions between brain areas and impairment in patients with nfvPPA/AOS, cognitive models were developed for apraxia of speech often based on language models, such as those proposed by Levelt [95] and aim to describe the processes involved in apraxia modeling the invariant and variant aspects of speech production [96,97].
